# Identification of GAD65 AA 114-122 reactive 'memory-like' NK cells in newly diagnosed Type 1 diabetic patients by HLA-class I pentamers

**DOI:** 10.1371/journal.pone.0189615

**Published:** 2017-12-13

**Authors:** Valentina Perri, Elena Gianchecchi, Loredana Cifaldi, Marsha Pellegrino, Ezio Giorda, Marco Andreani, Marco Cappa, Alessandra Fierabracci

**Affiliations:** 1 Type 1 Diabetes Centre, Infectivology and Clinical Trials Research Department, Bambino Gesù Children’s Hospital, IRCCS, Rome, Italy; 2 Pediatric Hematology and Oncology Department, Bambino Gesù Children’s Hospital, IRCCS, Rome, Italy; 3 Research Laboratories, Bambino Gesù Children’s Hospital, IRCCS, Rome, Italy; 4 Laboratory of Immunogenetics and Transplant Biology, IME Foundation, Polyclinic of Tor Vergata, Rome, Italy; 5 Endocrinology Department, Bambino Gesù Children’s Hospital, Rome, Italy; Baylor College of Medicine, UNITED STATES

## Abstract

Type 1 diabetes is an autoimmune disease, in which pancreatic β cells are destroyed by autoreactive T cells in genetically predisposed individuals. Serum beta cell autoantibody specificities have represented the mainstay for classifying diabetes as autoimmune-mediated and for stratifying risk in first-degree relatives. In recent years, approaches were attempted to solve the difficult issue of detecting rare antigen-specific autoreactive T cells and their significance to etiopathogenesis such as the use of the MHC multimer technology. This tool allowed the specific detection of increased percentages of GAD65 autoreactive T cells by means of HLA A*02:01 GAD65 AA 114–122 pentamers in newly diagnosed diabetics. Here we provide evidence that GAD65 AA 114–122 pentamers can depict a GAD65 AA114-122 peptide expandable population of functionally and phenotypically skewed, preliminary characterized CD3^-^CD8^dull^CD56^+^ ‘memory-like’ NK cells in PBMC of newly diagnosed diabetics. Our data suggest that the NK cell subset could bind the HLA class I GAD65 AA 114–122 pentamer through ILT2 inhibitory receptor. CD107a expression revealed increased degranulation of CD3^-^CD8^dull^CD56^+^ NK cells in GAD65 AA 114–122 and FLU peptide expanded peripheral blood mononuclear cells of diabetics following GAD65 AA 114–122 peptide HLA A*02:01 presentation in respect to the unpulsed condition. CD107a expression was enriched in ILT2 positive NK cells. As opposite to basal conditions where similar percentages of CD3^-^CD56^+^ILT2^+^ cells were detected in diabetics and controls, CD3^-^CD56^+^CD107a^+^ and CD3^-^CD56^+^ILT2^+^CD107a^+^ cells were significantly increased in T1D PBMC either GAD65 AA 114–122 or FLU peptides stimulated after co-culture with GAD65 AA 114–122 pulsed APCs. As control, healthy donor NK cells showed similar degranulation against both GAD65 AA 114–122 pulsed and unpulsed APCs. The pathogenetic significance of the CD3^-^CD8^dull^CD56^+^ ‘memory-like NK cell subset’ with increased response upon secondary challenge in diabetics remains to be elucidated.

## Introduction

Type 1 diabetes (T1D) is an autoimmune disease which results from destruction of the insulin-producing *β* cells present in the pancreatic islets of Langerhans [1]. This multifactorial disorder develops in human leukocyte antigen (HLA) genetically predisposed individuals with the contribution of still unknown environmental factors and stochastic events [2]. In the disease pathogenesis several immunotypes play important roles i.e. autoreactive CD4^+^ and CD8^+^ T cells, autoantibodies producing B lymphocytes and innate immunity components [3].

For long-time, combination screenings of autoantibodies (Abs) directed against insulin (IAA), proinsulin, glutamic acid decarboxylase (GAD) isoforms GAD65, GAD67, the insulinoma-associated antigen (IA-2)/tyrosine phosphatase-like molecule IA-2 *β* [4] have represented the mainstay for classifying diabetes as autoimmune-mediated and for stratifying risk in first-degree relatives [5]. Nevertheless, these immune markers are not directly pathogenetic as opposite to autoreactive T cells [3], consistent with the notion that before and by the time of clinical disease onset these cells have received antigen-specific stimulation [6].

Following several attempts in evaluating autoreactive T cell responses that lacked diabetes-specificity, the use of the major histocompatibility complex (MHC) multimer technology [3] was introduced to solve the difficult issue of detecting in the peripheral blood rare antigen-specific autoreactive T cells and pinpoint their significance to disease onset and progression. This tool allowed the specific detection of autoreactive T cells by means of HLA A*02:01 GAD65 AA 114–122 pentamers [6,7]. The possibility to detect these specialized cell populations would also offer the theoretical advantage of improved prediction strategies of disease as well as the opportunity to target them in immunomodulation therapies and foresee disease regression based on their physical disappearance or functional silencing [8].

Multimer tools could also nowadays foster investigations aimed to discover specific immunotypes of unexpected role in the disease pathogenesis. As regards, despite autoimmune conditions are indeed principally due to B and T lymphocytes, recent studies provide evidence that also natural killer (NK) cells play a significant role in the promotion and/or maintenance of altered adaptive immune responses; these cells are indeed involved in the establishment of peripheral tolerance and immune regulation in preventing disease onset [9,10]. In the pathogenetic process, NK cells realize cross-talk with dendritic cells (DCs) and T cells through different receptor-ligand interactions [11]. Despite NK cells are usually classified as a component of the innate immune system, they show characteristics of the adaptive immune system, such as clonal expansion of pathogen-specific cells, the generation of long-lasting ‘memory’ cells able to persist upon cognate antigen encounter, longevity as well as epigenetic modifications, and furthermore the possibility to induce an increased secondary recall response to re-challenge [12,13].

NK cells alterations have been reported in both T1D animal models and patients. In NOD (non-obese diabetic) mice, pancreatic NK cells with specific phenotype and a diminished functionality respect to spleen NK cells were identified allowing hypothesizing their putative involvement in the initiation of pancreatic insulitis [14]. Lehuen and colleagues (1988) reported that NKT cell number was able to influence significantly T1D onset since they observed the protective effect of NKT cell overexpression in V_14-J_281 transgenic NOD mice [15]. Furthermore, investigations have described a diminished NK cell number, altered lytic activity and changes in the expression of activating receptors in the peripheral blood of T1D subjects [16,17], whereas other studies highlighted the association between stage of disease and presence of transitory cellular differences [18,19].

Since NK cells do not undergo somatic gene rearrangements as occurs for T and B lymphocytes and do not express an unique antigen recognition receptor [20,21], specificity and diversity is allowed by the balance of a different array of surface activating and inhibitory NK cell receptors (NKRs) [22,23]. These characteristics can be influenced by aging and putatively disease development. When activatory signals prevail on inhibitory signals, proinflammatory cytokine production and perforin/granzymes-mediated target cytotoxicity [24] are triggered. The threshold of NK cell activation relies on cytokines; indeed, it is reduced in case of MHC class I molecule downregulation by transformed or infected cells [25–27]. Remarkably, inhibitory NKRs interact with classical MHC class I molecules expressed by all nucleated cells [28,29]. Interactions with myelomonocytic cells were found not unique to HLA-G but also to tetramers of other MHC class I molecules (including HLA-A*02:01) [30]. By using HLA A*0201-HCMV (human cytomegalovirus) pp65 tetramers, CD3^-^CD56^+^ NK cells were bound through their ILT2/LIR1 (immunoglobulin-like transcript 2 [ILT2], leukocyte immunoglobulin receptor B1 [LIR1]) expression [29]. Furthermore, lymphoid cells with a large proportion of CD3^-^CD56^+^ NK cells were stained with HLA-E tetramers at high frequency [31]. Of note, NK cells expressing inhibitory receptors are hyperresponsive to recognize and kill MHC class I expressing target cells since are deemed licensed [32] and educated [33]. Moreover, ILT2 molecules are expressed specially in CD56^dim^ NK cells, which are considered to have a cytotoxic function due to their capability to produce lytic granules, while they are not expressed in CD56^bright^ NK cells that perform regulatory activities for their ability to produce high amount of cytokines [27, 34]. In this manuscript, an improved gating strategy of Flow Cytometric analysis was implemented to pinpoint novel immune specificities by using HLA class I pentamers constructed with the GAD65 AA 114–122 epitope. Here we provide evidence that HLA-class I pentamers can depict a novel GAD65 AA 114–122 peptide expandable population of functionally and phenotypically skewed preliminary characterized ‘memory-like’ NK cells within an autoimmune condition. Few evidences have reported so far the existence of ‘memory-like’ NK cell subsets emerging upon viral infections or hapten-induced contact hypersensitivity [35,36,24]. The identified subset of GAD65 AA 114–122 reactive ‘memory-like’ NK cells might reveal potential undiscovered properties in T1D pathogenesis and development [12,13].

## Materials and methods

### Patients and controls

As part of an extended GAD65 AA 114–122 HLA class I pentamers analysis of PBMC from newly diagnosed diabetic patients recruited from Lazio region at the Endocrinology and Diabetes Unit at the Children's Hospital Bambino Gesù, Rome [37,38] a subgroup of 23 HLA-A*02:01 positive pediatric patients (10 males and 13 females, age of onset range 2.11 to 17.10, mean 10.58 years) was dedicated in this study to statistically evaluate the percentages of GAD65 pentamer reactive NK cells in diabetics *versus* 23 controls (Table 1) while 15 (5 males and 10 females, age of onset range 4.8 years to 15 years, mean 10.5 years) were dedicated to pentamer reactive NK cell phenotypical characterization (Table 1). PBMC from a group of 20 patients were also withdrawn during the follow-up from one to 3 years from disease onset (long-term disease) (10 males and 10 females, age at onset range 5.6 to 14.5, mean 10.7 years, (S1 Table)) to compare GAD65 pentamer reactive NK cell percentages respect to disease onset.

**Table 1:** Sex, age and diabetes-related autoantibodies profile (GAD65, IA2, IAA) in newly diagnosed T1D patients at disease onset used to define percentages of GAD65 pentamer reactive NK cells and their phenotypical characterization.

Newly diagnosed T1D patient	Sex	Age at onset (years,months)	GAD65 Abs (< 1 U/ml)	IA2 Abs (< 1.1 U/ml)	IAA (< 7%)
1*	F	12.1	**24**	1.1	2
2*^§^	F	13.9	**4.6**	0.1	4.6
3	F	6.9	**0.3**	0.1	**4.0**
4	M	10.6	**22**	**2.1**	**102**
5	F	2.6	0.6	**8**	**8**
6	M	2.11	0.6	0.8	**24**
7	F	17.8	**2**	**7.3**	5
8	M	17.10	**5.3**	**13**	**4**
9	F	12.3	**17**	0.4	**9**
10	M	7.1	**1.9**	**24**	5
11	F	4.9	**3.7**	**7**	**8.2**
12*^§§^	F	13	**19**	**10**	1
13*^§§§^	F	10.2	**1.5**	**4**	**14.5**
14	M	6.6	0.2	NT	**11**
15	F	11	**5**	**5.1**	5
16	M	5	**1.4**	**3.2**	**18**
17	M	14.11	**61.4**	**1.7**	**19.7**
18	M	13.3	0.4	0.6	**7**
19	M	13.8	**1**	0.6	6
20	F	9.3	**2.7**	**4**	6.5
21*	F	12.7	**1.6**	0.4	6
22*	F	14.2	**28**	**7.5**	5
23*	M	12.7	**13**	**10**	**9**
24*	F	5.8	**19**	**14**	**9.1**
25*	M	4.1	0.2	0.1	1
26*	F	6.9	0.3	0.1	4
27*	M	9.9	0.3	**11**	6
28*	F	10.4	**5**	**4.6**	**8.2**
29*	M	10.2	0.1	**4**	**9**
30*	M	6.4	**34**	**19**	**13**
31*	F	15	0.4	**40**	**7**

*Patients used for CD3^-^CD8^dull^ GAD65 pentamer reactive cells characterization.

^§^Associated celiac disease.

^§§^Associated thyroiditis, anti-gliadin, anti-transglutaminase Abs.

^§§§^Associated thyroiditis.

The patients’ sera were tested for Abs to glutamic acid decarboxylase (isoform 65) (GADA), protein tyrosine phosphatase insulinoma associated antigen 2 (IA2) and insulin (IAA) by radioimmunoassay (RIA), to thyroglobulin (Tg), thyroperoxidase (TPO) and transglutaminase (tTGA) by chemiluminescence (ADVIA Centaur analyzer, Siemens Healthcare, Germany) and to parietal cells (PCA) and the adrenal cortex (ACA) by indirect immunofluorescence (IFL).

Three T1D patients presented at disease onset associated autoimmune manifestations (Table 1). The control population included HLA-A*02:01 healthy blood volunteer donors (HD) recruited in our laboratory according to standard protocols [39]. They had no history of autoimmunity and no islet-related autoantibodies were detected in their serum. All enrolled patients and controls were unrelated.

The study was approved by the local Institutional Review Board (IRB) of Bambino Gesu`Children’s Hospital, regulating the use of human samples for experimental studies (Study protocol 170 CM/lb). Written informed consent was obtained from participants.

### PBMC isolation

Withdrawn PBMC samples were separated by Ficoll-Hypaque (Histopaque, Sigma-Aldrich Chemical Company, St Louis, MO, USA) from venous blood samples, according to standard procedures [37], and then frozen down in liquid nitrogen.

### HLA-A2 typing and subtyping

DNA was obtained from blood samples by a fully automated system (Maxwell, Promega, Milan, Italy). Low and high resolution typing for HLA-A locus was performed by polymerase chain reaction using sequence specific oligonucleotide (PCR-SSO) technique (Luminex, One Lambda, Canoga Park, CA, USA) or PCR with sequence specific primers (PCR-SSP, Olerup, Stockholm, Sweden).

### Stimulation of PBMC with the GAD65 AA 114–122 peptide

At the time of pentamer analysis cells were thawed and resuspended, at a density of 1x10^6^/ml in culture medium supplemented with GAD65 peptide AA 114–122 (VMNILLQYV, Proimmune Limited, Oxford, UK) at a concentration of 30 *μ*g/ml, for 4 days in 24-well round-bottomed plates (Falcon, Labware BD Biosciences, Oxnard, CA, USA) according to tetramer analysis established procedure [37]. As we already reported [37,38] Database search was performed with nonamers since class I binds peptides of 8–10 aminoacid residues and preferably nonapeptides. The GAD65 AA 114–122 nonapeptide had been selected for its high score (1080.239) of affinity binding to HLA A*02:01 in an HLA peptide motif search Database (http://www-bimas.cit.nih.gov/molbio/hla bind) (S2A and S2B Table) [38]. In addition to the peptide, PBMC were supplemented with interleukin-2 (IL-2, 25 IU/ml, Sigma-Aldrich). IL-2 was added to the cultures because we found that it improved cell survival in cryopreserved pathological samples without altering cell function [39].

As already described [37] in parallel experiments controls were set up by culturing PBMC from the same individual with IL-2 (25 IU/ml, Sigma-Aldrich) alone for 4 days, in place of the GAD65 AA 114–122 peptide plus IL-2, prior to the flow cytometry analysis (FACS) to ensure PBMC survival. At the end of the 4 days, PBMC, either stimulated with the GAD65 peptide plus IL-2 or incubated with IL-2 alone, were washed to remove the excess of GAD65 AA 114–122 peptide and IL-2 from the culture, so to maximally reduce the risk of nonspecific binding, when PBMC will subsequently be stained with labeled pentamers [37,38]. PBMC stimulated or not with GAD65 AA 114–122 peptide were cultured for additional 2 days with IL-2 (25 IU/ml) according to our established protocols [37,38].

### Cell staining with HLA A*02:01 GAD65 AA 114–122 peptide pentameric complexes

On day 6, following previously published protocols [37,38], approximately 5x10^5^ cells, stimulated with the GAD65 AA 114–122 peptide (supplemented with IL-2) or cultured with IL-2 alone, were washed and finally resuspended in approximately 50 *μ*l. 1 *μ*l of phycoerithrin (PE) labelled GAD65 AA 114–122 pentamer was added to each cell preparation and incubated in ice for 30 minutes in the dark, then washed in wash buffer. Mouse monoclonal antibody (mAb) anti-human CD8 at 1:20 dilution (fluorescein (FITC) labelled, clone HIT8a, cat# 555634, Becton & Dickinson (BD), Pharmingen, San Diego, CA, USA) and mouse mAb anti-human CD3 at 1:20 dilution (allophycocyanin (APC) labelled, clone UCHT1, cat# 555335, BD, Pharmingen) were added for further discriminating the different cell preparations. After staining, cells were immediately acquired for the analysis on the FACSCanto II (BD). Flow cytometric profiles were analyzed using the FACSDiva software (BD Bioscience). Dead cells were excluded from the analysis by side/forward scatter gating [39]. A minimum of twenty thousand events, gated on living cells, were collected per dataset.

### Control of specificity of GAD65 AA 114–122 pentamer reactive T cells

In parallel experiments, the same PBMC samples from both newly diagnosed diabetic patients and healthy controls were cultured with influenza virus (FLU) peptide (GILGFVFTL, 10μg/ml, Proimmune Limited) plus IL-2 (25 IU/ml, Sigma-Aldrich) and human immunodeficiency virus (HIV) peptide (SLYNTVATL, 10μg/ml, Proimmune Limited) plus IL-2 (25 IU/ml, Sigma-Aldrich). Cultures were carried out for 4 days, followed by IL-2 incubation for 2 days as above reported for the GAD65 AA 114–122 stimulation protocol. At the end of the incubation periods cell staining with HLA A*0201 GAD65 AA 114–122 pentameric complexes was carried out as above described (*see above*).

### Characterization of CD3^-^CD8^dull^CD56^+^ILT2^+^ GAD65 AA 114–122 pentamer reactive cells

A subsequent staining procedure for phenotypic characterization of CD3^-^CD8^dull^ GAD65 AA 114–122 pentamer reactive T cells was implemented by adding the following antibodies: mAb anti-human CD8 FITC (1:20, BD), mAb anti-human CD3 Alexa Fluor 700-A labelled (1:50, clone UCHT1, cat# 557943, BD), mouse mAb anti-human CD56 PE cyanine 7 (PECy7) labelled (clone B159, cat# 557747, BD) and mouse mAb anti-human ILT2/LIR1 (CD85J (Ig-like transcript (ILT)/leukocyte Ig-like receptor (LIR) (APC conjugated, clone HP-F1, cat# 17-5129-41, eBioscience) [29] both used at 1:50 dilution.

### GAD65 AA 114–122 pentamer reactive cells in long-term disease

By using the described procedure, GAD65 AA 114–122 pentamer reactive subsets were evaluated in PBMC of 20 long-term diabetic samples, in comparison with respective newly diagnosed samples, in a follow-up ranging from one to 3 years from disease onset (S1 Table).

### Functional evaluation of CD3^-^CD8^dull^CD56^+^ILT2^+^ GAD65 AA 114–122 pentamer reactive cells

#### GAD65 AA 114–122 peptide NK cell stimulation and activation in PBMC

Cryopreserved PBMC from newly diagnosed T1D patients were thawed and resuspended at a density of 1x10^6^/ml, in RPMI-1640 (GIBCO/BRL), supplemented with 2 mmol/l L-glutamine, 100 *μ*g/ml pen/strep, and 10% v/v FBS (Hyclone); cells were then cultured in the presence of the GAD65 AA 114–122 peptide at a concentration of 30 *μ*g/ml. Cells were cultured for 4 days in 24-well round-bottomed plates (Falcon) (1x10^6^ cells/well) according to the above described procedure (*see above*). In parallel experiments, control cell cultures were set up by incubating PBMC from the same patient with FLU peptide (GILGFVFTL, 10μg/ml, Proimmune Limited) plus IL-2 (25 IU/ml, Sigma-Aldrich). At the end of the 4 days, PBMC, either stimulated with the GAD65 AA 114–122 or FLU peptides plus IL-2 were washed in calcium-magnesium free 1X Dulbecco’s PBS (Euroclone) by centrifugation at 1500 rpm for 5 minutes at RT. The two sets of PBMC, either GAD65 AA 114–122 or FLU peptides stimulated, were cultured for additional two days in the same medium supplemented with IL-2 (100 IU/ml) in order to activate NK cells [40].

#### HLA A*02:01 antigen presenting cells pulsed with GAD65 AA 114–122 peptide

Cryopreserved EBV (Epstein Barr virus)-transformed antigen presenting cells (APCs) that had been generated, according to standard procedures, from HLA A*02:01 PBMC of newly diagnosed T1D patients [39] were thawed and resuspended in RPMI (GIBCO/BRL) supplemented with 2 mmol/l L-glutamine, 100 *μ*g/ml pen/strep and 20% FBS (Hyclone) and cultured for 5 days in T25 flasks (Falcon, Labware BD Biosciences). Cells were then rescued from flasks, pelleted at 1200 rpm for 5 minutes, resuspended in RPMI 2% FBS (Hyclone) medium, and plated at 1x10^6^/well in 24 well round bottomed culture plates (BD). Cells pulsed with GAD65 AA 114–122 peptide (30 *μ*g/ml) or unpulsed were left overnight at 26°C under sterile conditions.

#### Co-culture

On day 6 PBMC, either GAD65 AA 114–122 or FLU peptides stimulated for 4 days, additionally cultured for two days in the presence of IL-2 (100 IU/ml), were co-cultured with pulsed or unpulsed APCs at 1:3 (PBMC:APCs) ratio in 96 well round bottomed culture plates (Corning Incorporated, Corning, NY 14831–001, USA) for 2 and half hours in RPMI 10% FBS complete medium (*see above*) additionally supplemented with GolgiStop reagent (1:500 dilution, BD Biosciences). An experimental positive control was set-up by NK cell isolation from PBMC of a HD volunteer previously obtained with the RosetteSep method (Stem Cell Technologies, Vancouver, Canada) and FicollPaque Plus (Lympholyte, Cedarlane Laboratories, Burlington, Ontario, USA). Isolated NK cells have been routinely checked for the percentage of CD3^-^CD56^+^ cells by FACS analysis and those with purity greater than 90% were cultured with 200 IU/ml of recombinant human IL-2 (Sigma-Aldrich) at 37°C and used up to 5 days after isolation as effectors in degranulation assay. Isolated NK cells were then co-cultured with K562 cells (American Type Culture Collection, ATCC), a tumoral cell line known to induce NK cell degranulation according to standard protocols, as control target [41], and with either GAD65 AA 114–122 peptide pulsed and unpulsed APCs.

#### Detection of NK cell degranulation

Degranulation assay of NK cells following GAD65 AA 114–122 peptide presentation was performed through quantification of cell-surface CD107a expression by FACS analysis [40]. Briefly, at the end of the co-culture period, culture plate was centrifuged at 2000 rpm for 2 minutes and cell staining was directly performed by adding the mixture of mouse mAbs anti- human CD3 Alexa Fluor 700-A (1:50, BD), CD56 PECy7 (1:50, BD), CD8 PECy5 (1:30, clone RPA-T8, cat# 557746, BD), ILT2 (APC,1:50, eBioscience) and CD107a FITC (1:10, clone H4A3, cat# 555800, BD).

### Correlation of GAD65 AA 114–122 pentamer reactive cells with markers of diabetes metabolic control and presence of circulating diabetes-related Abs

An initial evaluation of the functional significance of the identified GAD65 AA 114–122 pentamer reactive cell population and its phenotypically characterized counterpart in diabetic patients was conducted through correlation of their percentage with metabolic markers of disease i.e. glycated hemoglobin (HbA1c), C-peptide, lipemic profile (including cholesterol (mg/dl), HDL (mg/dl), LDL (mg/dl) and triglyceride (mg/dl) values) and need for exogenous insulin (expressed as U/kg/die) at disease onset.

Furthermore, significance to etiopathogenesis was initially assessed through correlation of GAD65 AA 114–122 pentamer reactive cell percentages with titers of circulating islet-related autoantibodies i.e. anti-GAD65, anti-IA2 and IAA.

### Statistical analysis

To calculate absolute values of GAD65 AA 114–122 pentamer reactive cells, CD3^+^CD8^bright^, CD3^+^CD8^dull^, total CD3^+^CD8^+^, CD3^-^CD8^dull^ and CD3^-^CD8^dull^CD56^+^ILT2^+^ respective percentages detected after GAD65 AA 114–122 peptide stimulation were subtracted of corresponding values obtained under IL-2 stimulation alone. Percentages in T1D patients *versus* (vs) controls were then analyzed for statistical significance with a two-tailed Student t test (Mann-Whitney (MW) test, paired and Unpaired *t* test with Welch’s correction). Normal distribution was tested using Kolmogorov-Smirnov (KS) test. A Fisher’s exact test was computed for 2x2 tables. The correlation coefficients between percentages of CD3^-^CD8^dull^GAD65 pentamer reactive cells and metabolic parameters i.e. HbA1c, C-peptide, insulin requirement, lipemic profile or autoantibodies levels were evaluated with Spearman test. Results were analyzed by using the GraphPad Prism software version number 5 (San Diego, CA, USA). A result with p <0.05 was considered statistically significant.

## Results

### Detection of GAD65 AA 114–122 peptide reactive cells

**Fig 1:**
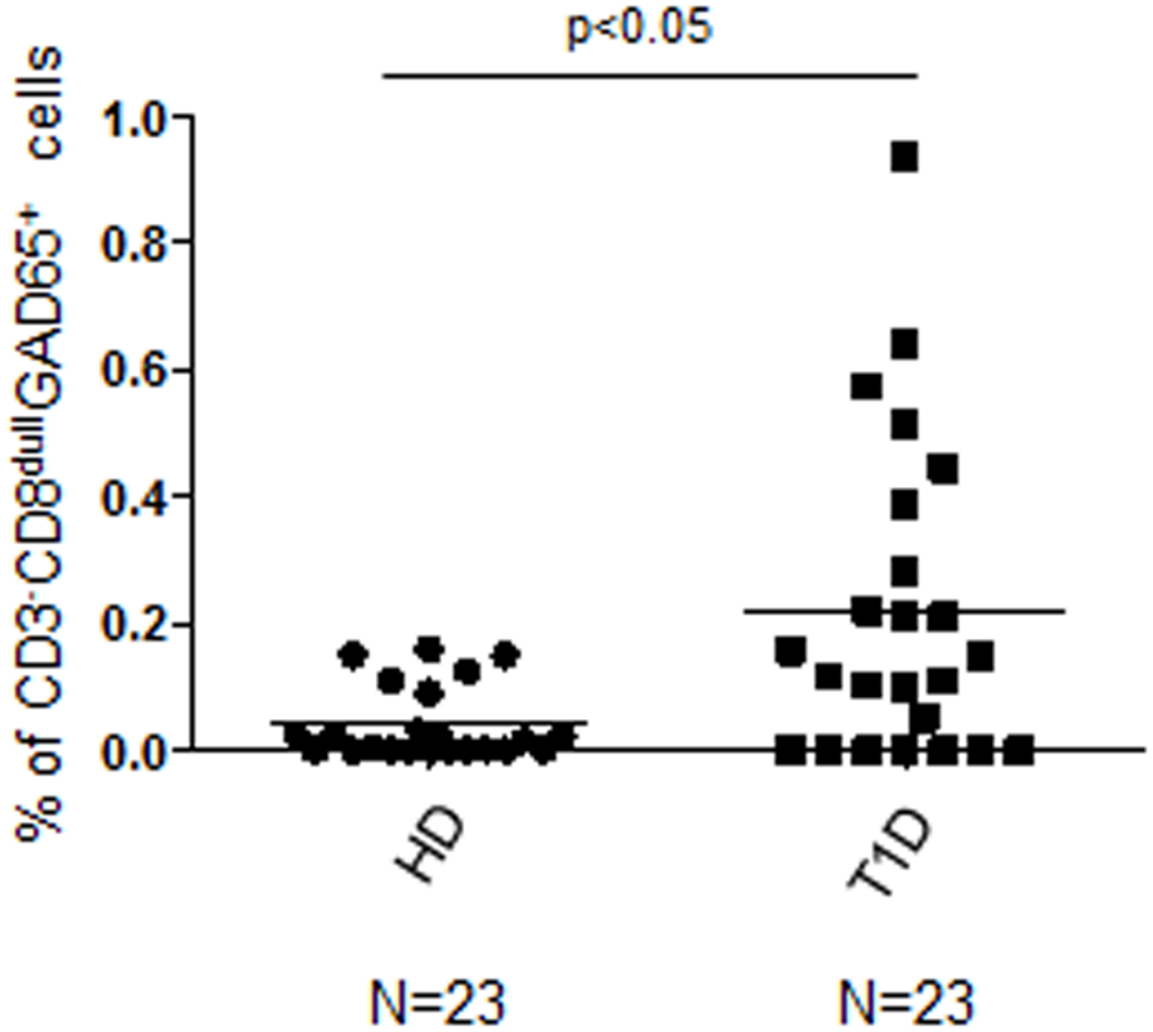
Increased percentage of CD3^-^CD8^dull^GAD65 AA 114–122 pentamer reactive cells after GAD65 AA 114–122 peptide stimulation in T1D patients compared to healthy controls. PBMC from healthy blood donors and T1D patients, were GAD65 AA 114–122 peptide stimulated, stained with CD3, CD8 and GAD65 AA 114–122 HLA A*02:01 pentamers, then analyzed by FACS to determine the percentages of GAD65 pentamer reactive cells in CD3^+^CD8^bright^, CD3^+^CD8^dull^, total CD3^+^CD8^+^ and CD3^-^CD8^dull^ subsets (see S1 Fig). In the graph, horizontal bars represent the mean percentage of CD3^-^CD8^dull^ GAD65 AA 114–122 reactive cells and each symbol represents an individual: circle dots represents the normal and square dots the diabetic. Percentages of CD3^-^CD8^dull^ GAD65 reactive cells refer to analyzed events within flow-cytometry gates as shown in representative dot plots in S1 Fig. GAD65^+^ = reactive with PE-labelled GAD65 AA 114–122 pentamer. N = number of T1D patients and HD controls analyzed.

By using an improved gating strategy of Flow Cytometry analysis in respect to previous attempts [37], aiming to discriminate CD8^bright^/CD8^low^ cell subsets, we detected, in an extended group of newly diagnosed T1D patients compared to healthy controls, a significant increase in the percentage of GAD65 AA 114–122 pentamer reactive CD3^+^CD8^bright^ T cells (S1 and S2A Figs, KS test p<0.05; Unpaired t test with Welch's correction p = 0.0210), CD3^+^CD8^dull^ T cells (S1 and S2B Figs, KS test p<0.05; Unpaired t test with Welch's correction, p = 0.0312), total CD3^+^CD8^+^ T cells (S2C Fig, KS test p<0.05; Unpaired t test with Welch’s correction p = 0.0313) and CD3^-^CD8^dull^ cells (S1 Fig; Fig 1, KS test p<0.05; Unpaired t test with Welch’s correction p = 0.0023; S2D Fig, KS test p<0.05; Unpaired t test with Welch's correction p = 0.0037). Specificity of GAD65 AA 114–122 reactive T cells was confirmed in experiments of GAD65 AA 114–122 pentamer staining in PBMC of patients stimulated with the same GAD65 AA 114–122 peptide (GAD65). Conversely, no significant reactivity was observed after stimulating the same PBMC samples with FLU or HIV peptides (S3A Fig, CD3^+^CD8^bright^: KS test p<0.05; MW test GAD65 (GAD) vs FLU stimulated p = 0.0027, MW test GAD65 vs HIV p = 0.0008; S3B Fig, CD3^+^CD8^dull^: KS test p<0.05; MW test GAD65 vs FLU p = 0.0004, MW test GAD65 vs HIV p = 0.0001; S3C Fig, total CD3^+^CD8^+^: KS test p<0.05; MW test GAD65 vs T1D FLU p = 0.0004, MW test GAD65 vs HIV p = 0.0001; S3D Fig, CD3^-^CD8^dull^: KS test p<0.05; MW test GAD65 vs FLU p = 0.0048, MW test GAD65 vs HIV p = 0.0005).

**Fig 2:**
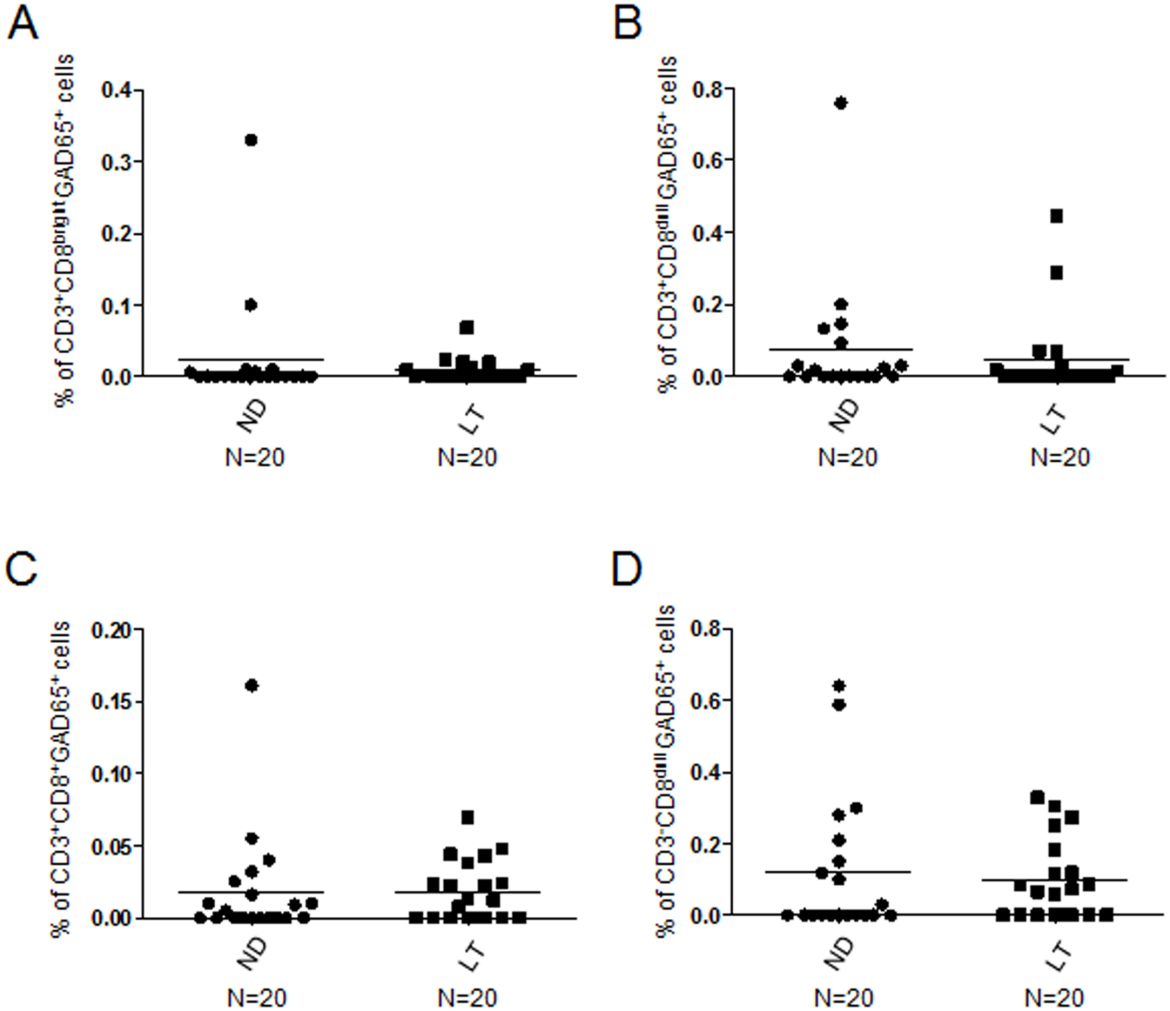
Similar percentages of GAD65 AA 114–122 HLA A*0201 pentamer reactive cells are circulating in newly diagnosed vs long-term T1D patients. Relative percentages of CD3^+^CD8^bright^ (A), CD3^+^CD8^dull^ (B), total CD3^+^CD8^+^ (C) and CD3^-^CD8^dull^ cells (D) in PBMC of newly diagnosed (ND, circle dots) vs long-term T1D patients (LT, square dots); horizontal bars, average values are reported.

In a follow-up period of one to three years from diagnosis there was no significant differences in the percentages of all GAD65 AA 114–122 reactive subsets in long-term diabetic patients (S1 Table) vs newly diagnosed diabetics (Fig 2A, CD3^+^CD8^bright^: KS test p<0.05; Unpaired t test with Welch’s correction p>0.05; Fig 2B, CD3^+^CD8^dull^: KS test p<0.05; Unpaired t test with Welch’s correction p>0.05; Fig 2C, total CD3^+^CD8^+^: KS test p<0.05; Unpaired t test with Welch’s correction p>0.05; Fig 2D, CD3^-^CD8^dull^: KS test p<0.05; Unpaired t test with Welch’s correction p>0.05).

### Characterization of CD3^-^CD8^dull^CD56^+^ILT2^+^ GAD65 AA 114–122 pentamer reactive cells

**Fig 3:**
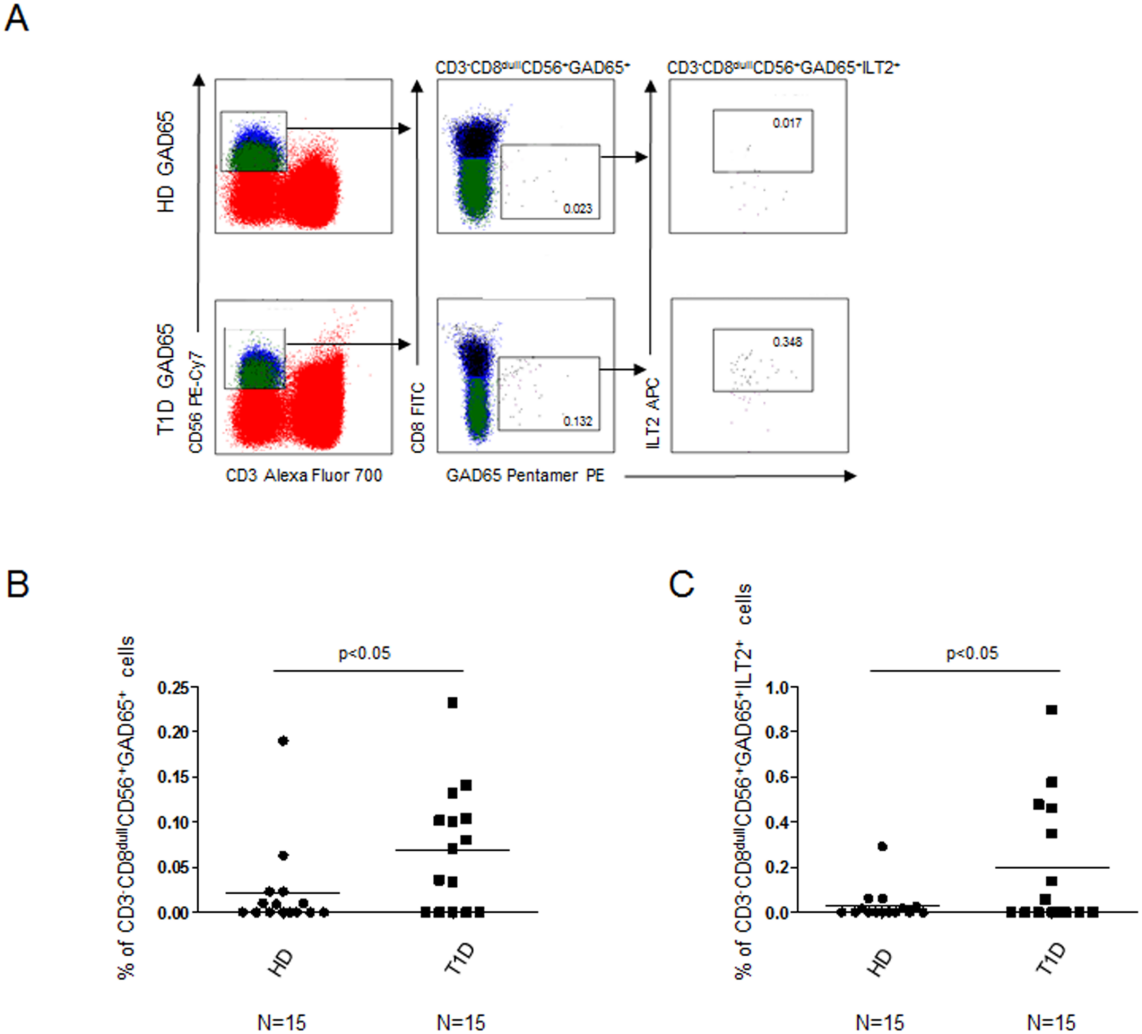
Increased percentages of CD3^-^CD8^dull^CD56^+^ and CD3^-^CD8^dull^CD56^+^ILT2^+^GAD65 AA 114–122 pentamer reactive cells in T1D patients. Relative percentages of CD3^-^CD8^dull^CD56^+^ and CD3^-^CD8^dull^CD56^+^ILT2^+^ GAD65 AA 114–122 pentamer reactive cells in T1D patients vs healthy controls after GAD65 peptide stimulation. (A) Representative experiment showing the flow-cytometry gate strategy; (B) Summary of results of CD3^-^CD8^dull^CD56^+^ GAD65 AA 114–122 pentamer reactive cells obtained in 15 healthy donors (circle dots) and 15 T1D patients (square dots); (C) Summary of results of CD3^-^CD8^dull^CD56^+^ ILT2^+^ GAD65 AA 114–122 pentamer reactive cells. Horizontal bars, average values are reported.

Further characterization of the CD3^-^CD8^dull^ subset evidenced that percentages of CD3^-^CD8^dull^ CD56^+^ GAD65 AA 114–122 pentamer reactive cells were significantly higher in T1D patients than in controls (Fig 3A and 3B, KS test p<0.05; Unpaired t test with Welch's correction p = 0.0411). CD56 positivity confirmed the NK cell subset identity.

Moreover, percentages of CD3^-^CD8^dull^CD56^+^ILT2^+^ NK cells were significantly increased in T1D patients vs controls (Fig 3A and 3C, KS test<0.05; Unpaired t test with Welch's correction, p = 0.0461) indicating that in T1D samples, the NK cell subset preferentially binds the HLA class I GAD65 AA 114–122 pentamer through ILT2 receptor.

### PBMC evaluation of CD3^-^CD56^+^ILT2^+^ NK cells

**Fig 4:**
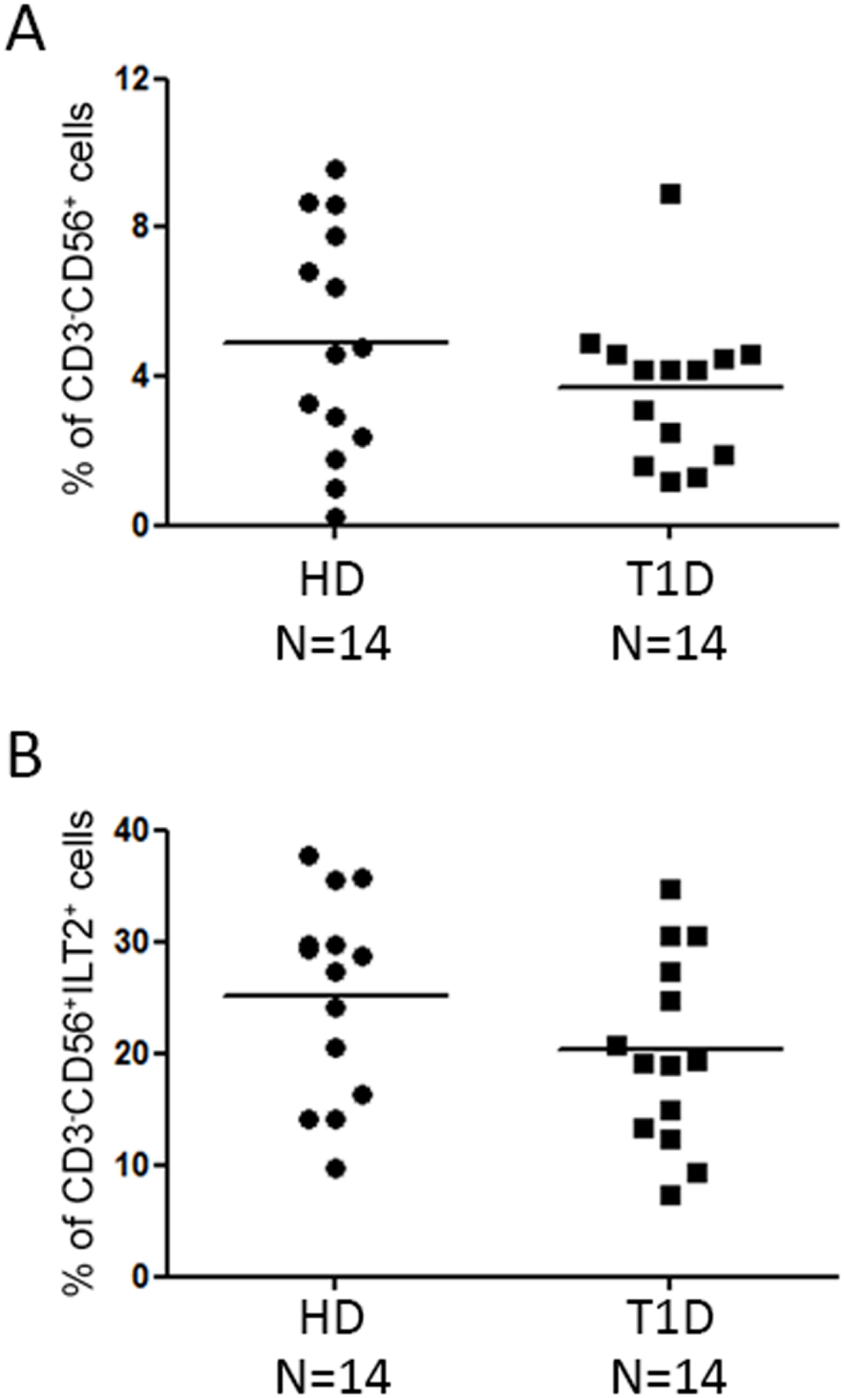
Similar percentage of total and ILT2 expressing NK cells between healthy donors and T1D patients. The percentage of CD3^-^CD56^+^ cells (A) and of CD3^-^CD56^+^ILT2^+^ cells (B) were assessed by flow cytometric analyses of IL-2 alone treated PBMC. NK cell phenotype of 14 healthy donors (HD, circle plots) was compared with that of 14 T1D patients (square dots); horizontal bars, average values are shown. No significant differences are reported (KS test, unpaired t test).

In order to exclude that the increase of GAD65 AA 114–122 pentamer reactive cells in T1D patients, compared to healthy donors, could be associated with the different amount of ILT2 expressing NK cells, cytofluorimetric analysis of CD3^-^CD56^+^ILT2^+^ cells in T1D and HD samples was performed. Percentages of CD3^-^CD56^+^ cells were similar between IL-2 alone treated PBMC of T1D patients vs controls (Fig 4A, KS test p>0.10; Unpaired t test p = 0.2243). No differences of percentages of CD3^-^CD56^+^ILT2^+^ NK cells were also observed in T1D patients vs controls (Fig 4B, KS test p>0.10; Unpaired t test p = 0.1394). These data suggest that the increased percentage of ILT2^+^GAD65 AA 114–122 pentamer reactive NK cells could be related to the pathophysiological features of the disease.

### Degranulation assay of CD3^-^CD56^+^ILT2^+^ NK cells

Finally, degranulation assay was performed to investigate the function of ILT2^+^GAD65 AA 114–122 reactive NK cells.

**Fig 5:**
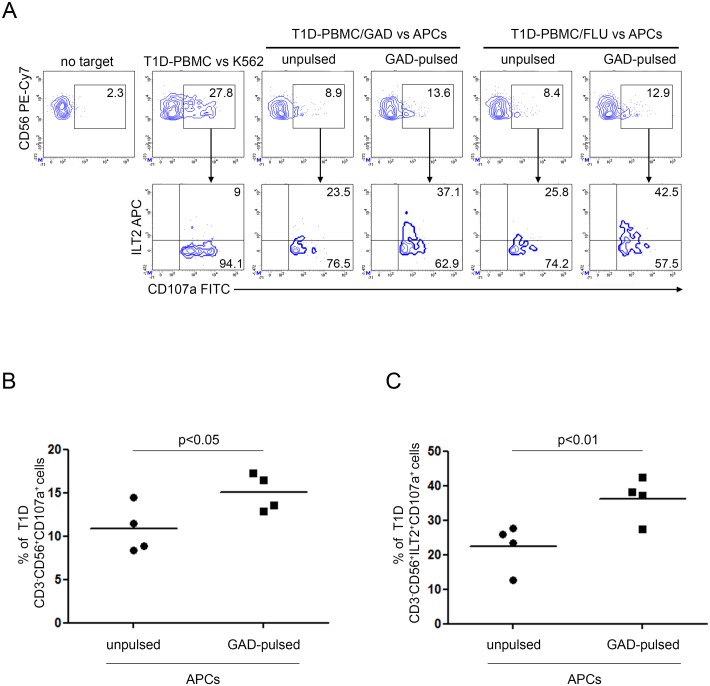
Increased susceptibility of GAD65 AA 114–122 peptide-pulsed APCs to T1D NK cell-mediated recognition associates with NK cell-ILT2 expression. Degranulation of CD3^-^CD56^+^ILT2^+^ NK cells of PBMC from T1D patients, expanded with GAD65 AA 114–122 or FLU peptides, measured as CD107a cell-surface expression following stimulation with APCs, either left unpulsed or GAD65 AA 114–122 peptide-pulsed. (A) A representative experiment out of two performed is shown. K562 cells were used as positive control. The percentage of CD3^-^CD56^+^CD107a^+^ NK cells (upper panel) and CD3^-^CD56^+^CD107a^+^ILT2^+^ (lower panel) is indicated for each condition. (B) Summary of CD3^-^CD56^+^CD107a^+^ and (C) CD3^-^CD56^+^ILT2^+^CD107a^+^ NK cell percentage of four T1D PBMC, expanded with GAD65 AA 114–122 or FLU peptides, following stimulation with APCs, either left unpulsed (circle dots) or GAD65 AA 114–122 (GAD65)-pulsed (square dots); horizontal bars, average values are reported.

**Fig 6:**
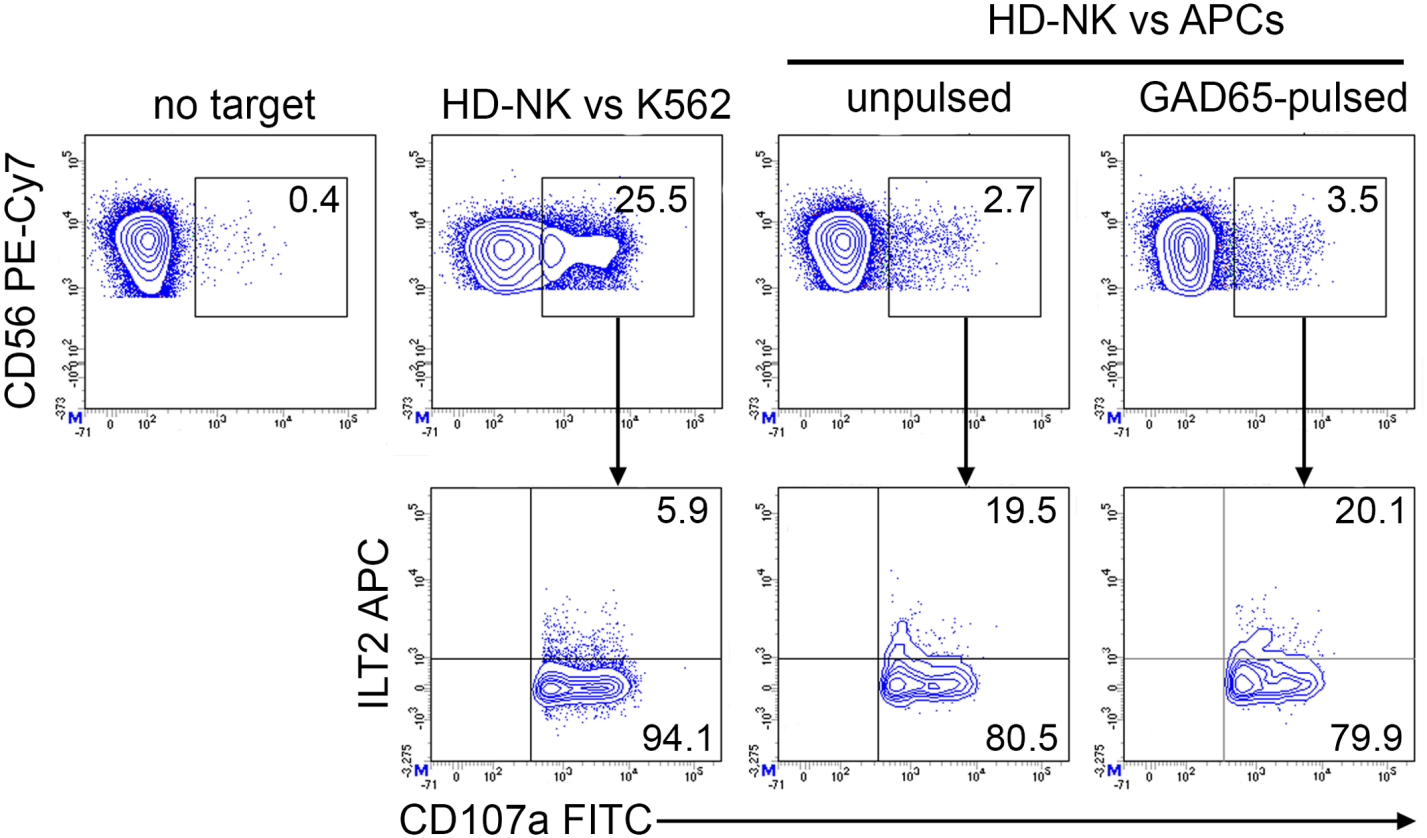
Unchanged healthy donor NK cell degranulation in response to GAD65 AA 114–122 peptide-pulsed APCs. Degranulation of CD3^-^CD56^+^ILT2^+^ NK cells of PBMC from HD patients, expanded with GAD65 AA 114–122 peptide, measured as CD107a cell-surface expression following stimulation with APCs, either left unpulsed or GAD65 AA 114–122 peptide-pulsed. A representative experiment out of two performed is shown. K562 cells were used as positive control. The percentage of CD3^-^CD56^+^CD107a^+^ NK cells (upper panel) and of CD3^-^CD56^+^CD107a^+^ILT2^+^ (lower panel) is indicated for each condition.

CD107a staining revealed increased degranulation of CD3^-^CD8^dull^CD56^+^ NK cells in GAD65 AA 114–122 and FLU peptide expanded PBMC of diabetic patients following GAD65 AA 114–122 peptide HLA A*02:01 APCs presentation in respect to the unpulsed condition (Fig 5A, upper panel). CD107a expression was enriched in ILT2 positive NK cells from both GAD65 and FLU peptide expanded PBMC in response to GAD65 AA 114–122 pulsed APCs (Fig 5A, lower panel). Percentages of CD3^-^CD56^+^CD107a^+^ cells were significantly increased in T1D PBMC either GAD65 AA 114–122 or FLU stimulated after co-culture with GAD65 AA 114–122 pulsed APCs (Fig 5B, paired t test p = 0.0131). Percentages of CD3^-^CD56^+^ILT2^+^CD107a^+^ cells were also significantly increased in T1D PBMC either GAD65 AA 114–122 or FLU stimulated after co-culture with GAD65 AA 114–122 pulsed APCs (Fig 5C, paired t test p = 0.0017). As control, T1D PBMC showed higher degranulation following stimulation by K562 cells (Fig 5A, upper panel). Moreover, since K562 cells are negative for MHC class I molecules, no ILT2 enrichment was detected (Fig 5A, lower panel). Differently, healthy donor NK cells showed similar pattern of degranulation against both HLA-A*02:01 GAD65 AA 114–122 pulsed and unpulsed APCs (Fig 6).

### Correlation of GAD65 pentamer reactive cells with diabetes markers of metabolic control

**Fig 7:**
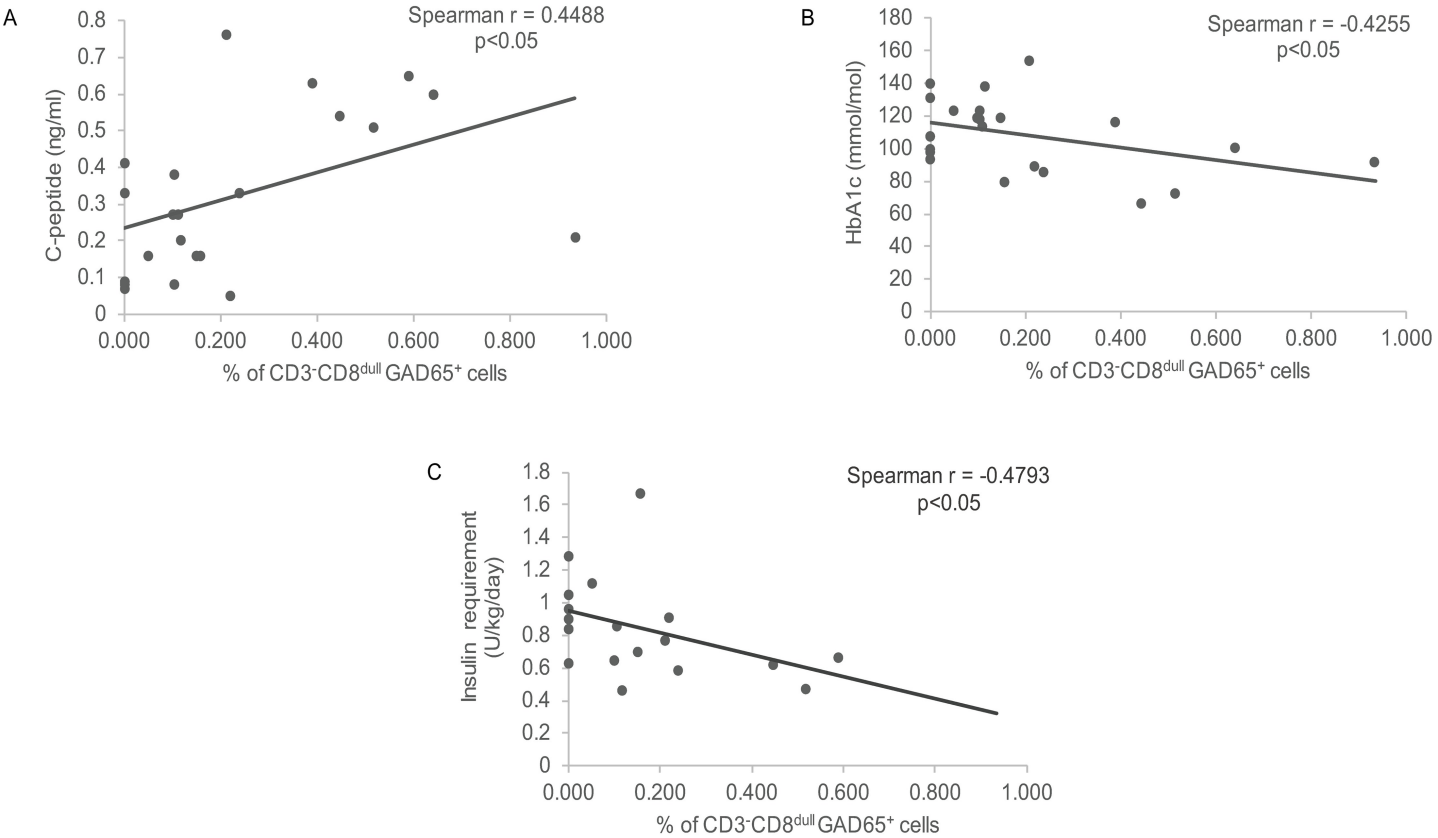
Correlation of GAD65 pentamer reactive cells with metabolic markers. (A) Direct correlation with C-peptide values; (B) Inverse correlation with HbA1c values; (C) Inverse correlation with insulin requirements.

In evaluating the interrelationship between percentages of CD3^-^CD8^dull^GAD65 pentamer reactive cells and metabolic markers of disease a direct correlation was found with C-peptide concentration (r = 0.4488, p = 0.0317) (Fig 7A) while an inverse correlation was detectable with HbA1c values (r = -0.4255, p = 0.0483) and insulin requirements (r = -0.4793, p = 0.0441) in the population of newly diagnosed diabetics indicative of a protective effect (Fig 7B and 7C). Conversely no correlation was found between percentages of CD3^-^CD8^dull^GAD65 pentamer reactive cells and markers of lipemic control i.e. total cholesterol (r = -0.1315, p = 0.6148), HDL (0.001397, p = 0.9964), LDL (r = -0.4185, p = 0.1547) and triglycerides (r = 0.01503, p = 0.9543) (S4 Fig).

### Correlation of GAD65 pentamer reactive cells with circulating diabetes-related Abs levels

**Fig 8:**
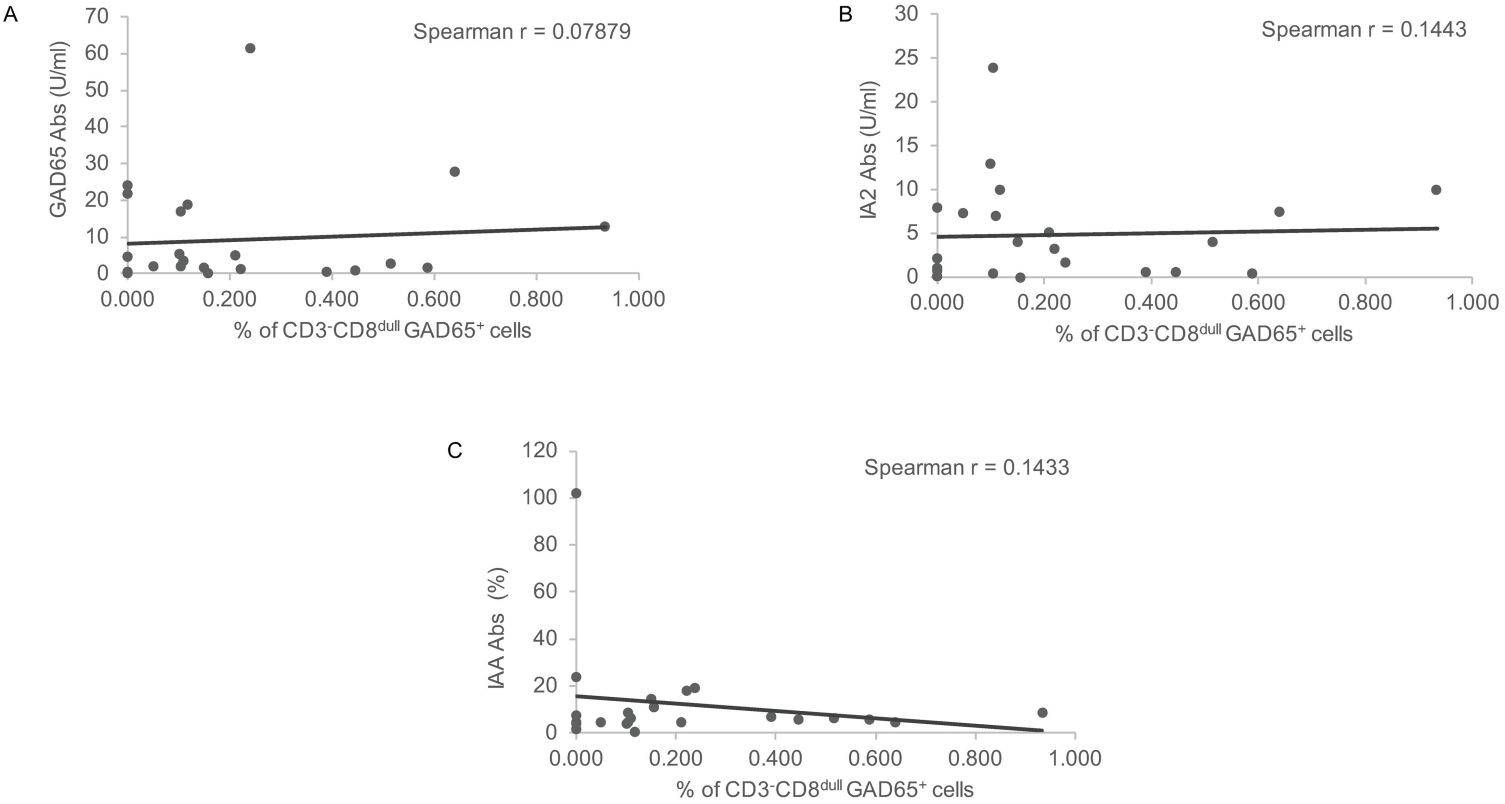
Correlation of GAD65 pentamer reactive cells with Abs levels. (A) No correlation with GAD65 Abs; (B) No correlation with IA2 Abs; (C) No correlation with IAA.

No correlation was found between percentages of CD3^-^CD8^dull^GAD65 pentamer reactive cells and circulating autoantibodies levels (Fig 8A, 8B and 8C) i.e. GAD65 Abs (r = 0.07879, p = 0.7208), IA2 Abs (r = 0.1443, p = 0.5219) and IAA (r = 0.1433, p = 5142), the last being of unknown significance to disease pathogenesis.

## Discussion

The application of multimer technology in detecting autoantigen specific T cell responses, could be of upmost relevance in investigating disease pathogenesis. Indeed, these markers modify more rapidly than autoantibodies that are secreted by long-lived plasma cells. Remarkably, antigen-specific immune monitoring could provide earlier endpoints than metabolic markers in assessing promptly whether the aimed immunedeviation has been achieved, since clinical endpoints, i.e. HbA1c values and insulin needs, are under the influence of diabetologists interventions, lifestyle and insulin sensitivity [8]. In this manuscript we provide evidence, by means of an extended analysis of T1D patients and healthy controls, that multimer technology can offer the additional advantage of identifying peculiar antigen-reactive subpopulations, preliminary characterized, such as the CD3^-^CD8^dull^CD56^+^ GAD65 AA 114–122 pentamer reactive ‘memory-like’ NK cells after a short GAD65 AA 114–122 peptide antigenic expansion. These cells were present at increased percentage in the peripheral blood of newly diagnosed T1D patients in respect to healthy controls. Remarkably, in long-term diabetics a secondary recall response to GAD65 AA 114–122 peptide stimulation of this subset was detected, suggesting their long-term survival thus confirming a characteristic feature of the adaptive immune system.

As stated in the Introduction, analysis of the frequency and activation state of NK cells in human T1D patients, carried out in different stages of disease, produced so far contrasting results with studies underlying that the disease onset is marked by a slight reduction in blood NK cells; conversely, these are unusually activated in some patients with interferon gamma (IFN-γ) expression [42]. Putatively, because of prolonged hyperglycemia, NK cells exhibited a markedly lower expression of NK p30/p46 cell activating receptor molecules compared to control subjects. Decreased expression of NKG2D was detected in diabetic patients independently of disease-duration. T1D patients had also an increased frequency of KIR gene haplotypes including KIR2DS3 gene underlying a genetic interaction between KIR and HLA complexes [42].

Thus, in unraveling the pathophysiological role of the discovered putative ‘memory-like’ NK cell subset, we attempted an initial characterization of this subset. ILT2 molecule expression was enriched in GAD65 AA 114–122 pentamer reactive NK cells in diabetic patients suggesting an higher affinity for HLA A*02:01 molecule. Remarkably, our experiments showed that CD3^-^CD56^+^ NK cells circulate in similar percentages in the peripheral pool of T1D patients and healthy controls. In addition, they exhibit similar expression levels of ILT2 [43–46]. Degranulation of CD3^-^CD56^+^ NK cells was detected following PBMC expansion with either GAD65 AA 114–122 or FLU peptide then challenged with HLA A*02:01 GAD65 peptide-pulsed APCs. Interestingly, CD107a expression was found enriched in ILT2 positive cells. As control condition, no GAD65 AA 114–122 specific degranulation was detectable in HD NK cells challenged with GAD65 AA 114–122 peptide-pulsed APCs. This degranulation activity is indicative of a level of cytotoxicity that does not exclude for the same subset a regulatory function possibly exerted in case a particular balance of activating/inhibitory receptors is manifested in the stage of disease where autoreactive T cells are expanded and activated.

Preliminary data show significant correlation between percentages of CD3^-^CD8^dull^ GAD65 pentamer reactive cells and metabolic control markers of disease i.e. C-peptide, while an inverse correlation is found with HbA1c levels and insulin requirements in newly diagnosed T1D patients, thus inferring to a protective role of the subset. In the attempt to unravel the underlying mechanism, CD3^-^CD56^+^ILT2^+^CD107a^+^ cells could exert their putative protective effect through cytotoxic properties against GAD65 APCs [47]. The NK cell-mediated clearance of this subset could affect autoantigen presentation to T cells whose reactivity is involved in Type 1 diabetes pathogenesis thus implying for GAD65 pentamer reactive NK cells a protective function. Of note, no correlation was found between CD3^-^CD8^dull^ GAD65 pentamer reactive cells and diabetes-related Abs the last being not directly pathogenetic as opposite to autoreactive T cells.

Nevertheless, these data and speculative observations do not provide full exploitation of the functional significance of this NK subset that requires to be evaluated in larger experimental settings. Definitively, in addition to investigating receptors expression, a full functional assessment would also require extensive evaluation of NK cell cytokine production at different stages of activation. Remarkably, NK cells are known to play important roles in determining immune responses not only through their cytolytic functions but also through their capacity to secrete cytokines and express chemokine receptors. As regards the ILT2-dependent interaction of memory–like NK cells with HLA A*0201 GAD65 pentamers, it is important to recall that HLA class I is indeed involved in regulating the production of cytokines through ILT2/CD85J binding [48]. In particular, blocking ILT2/CD85J-HLA class I interaction increased the release of IFN-γ in NK/immature DCs cocultures [48].

On a speculative basis, although the significance of the putative ‘memory-like’ NK cell subsets still awaits to be fully exploited and validated, their appearance was reported even upon viral infections such as HCMV. Although these NK cells have always been considered short-lived innate lymphocytes they are indeed able of long-lasting, antigen-specific memory to haptens and viruses [35,36,24]. In addition, cytokine stimulation alone can induce functional memory-like NKs with longevity [49]. As regards their characterization, limited data are available from literature. HCMV infection in particular was shown to shape the NK cell receptor repertoire inducing a specific CD94/NKG2C^+^ NK cell subset expressing the CD57 protein of terminal differentiation. This subset displays the highly differentiated surface CD56^dim^CD16^bright^LIR-1^+^KIR^+^NKG2A^-^ phenotype with self-KIRs (variable killer cells immunoglobulin like receptors) expression [50]. Lower levels of natural cytotoxicity receptors (NCRs) i.e. NKp30 and NKp46 were found on their surface while higher expression of CD85j that specifically recognizes HLA class I were detected. Decreased expression of FcɛRγ (high affinity immunoglobulin epsilon receptor subunit γ), SYK (spleen tyrosine kinase) and EAT-2 (Ewing's sarcoma-associated transcript 2) adaptor molecules also affect functional changes of adaptive NKs [51,52]. Several evidences suggest that different NK cell subsets act in a different manner against distinct pathogens. This was demonstrated by the fact that Ly49H+ and Ly49H- subsets exert complementary protective effects to murine cytomegalovirus since generating memory and cytokine-activated NK cells respectively [53]. Increase of CD56^dim^/CD16^bright^/NKG2C^+^ NK cells were also discovered in patients affected by other viral infections such as hepatitis C (HCV), hepatitis B (HBV) [54,55], Epstein Barr (EBV) [56] or human immunodeficiency virus (HIV)-1 [57]. Similarly to hapten-induced contact hypersensitivity, influenza virus-induced memory NK cells also reside in mice liver and are restricted to CD49a^+^DX5^-^ NKs; this subset was found to remember encountered antigen after primary infection and were more protective upon subsequent infection [58]. Furthermore, NK cells were discovered to play dual roles during influenza virus infection, conferring either pathological damage or protection according to their load [59,60]. Additional evidence confirms that memory-like NK cells survive long-term, with expansion depending on IL-21, playing a role in vaccine-induced protective immunity against a bacterial pathogen as *Mycobacterium tuberculosis* [61].

In conclusion, this report is the first original demonstration of the presence of an unexpected, preliminary phenotyped ‘memory-like NK subset’ with increased response upon secondary challenge in an autoimmune condition i.e. in T1D patient’s vs healthy controls. GAD65 AA 114–122 peptide VMNILLQYV appears as a key driving force regulating the differentiation of a functionally and phenotypically skewed NK cell subset. Their putative pathogenetic significance to T1D pathogenesis remains to be elucidated such as the expression and concerted functions of their surface activating/inhibitory receptors with special reference to the onset of the autoimmune disease. Remarkably, on a speculative basis, as demonstrated in response to pathogens [61], this subset can be variably affected during established disease through interaction with other immunotypes. Our preliminary functional evaluation interestingly envisages that memory-like NKs can be expanded under autoantigen-non specific bystander conditions as viral infections.

## Supporting information

S1 Fig FACS gating strategy to analyze different GAD65 AA 114–122 AA 114–122 pentamer reactive subsets after GAD65 peptide plus IL-2 or IL-2 incubation alone. Representative dot plot analysis showing GAD65 AA 114–122 HLA A*02:01 pentamer reactivity on peripheral blood lymphocytes of a T1D patient and a healthy control gated on CD3^+^CD8^bright^, CD3^+^CD8^dull^ and CD3^-^CD8^dull^ cells. The percentage of GAD65 AA 114–122 pentamer reactivity is indicated for each condition.
(TIF)

S2 Fig Increased percentages of GAD65 AA 114–122 HLA A*02:01 pentamer reactive cells in T1D patients vs healthy controls. Relative frequencies of CD3^+^CD8^bright^ (A), CD3^+^CD8^dull^ (B), total CD3^+^CD8^+^ (C) and CD3^-^CD8^dull^ (D) GAD65 AA 114–122 pentamer reactive cells in T1D patients (square dots) vs healthy controls (circle dots) after GAD65 AA 114–122 peptide stimulation; horizontal bars, average values are reported. Percentages refer to analyzed events within flow-cytometry gates as shown in representative dot plots in S1 Fig.
(TIF)

S3 Fig Specific reactivity to GAD65 AA 114–122 HLA A*02:01 pentamer. Relative percentages in T1D PBMC of CD3^+^CD8^bright^ (A), CD3^+^CD8^dull^ (B), total CD3^+^CD8^+^ (C) and CD3^-^CD8^dull^ (D) GAD65 AA 114–122 pentamer reactive cells after stimulation with GAD65 AA 114–122 peptide (square dots) vs FLU (triangle dots) and HIV peptide (open circle dots); horizontal bars, average values are shown.
(TIF)

S4 Fig Correlation of GAD65 pentamer reactive cells with metabolic markers. (A) No correlation with total cholesterol levels; (B) No correlation with HDL levels; (C) No correlation with LDL levels; (D) No correlation with triglycerides levels.
(TIF)

S1 Table Sex, age and diabetes-related autoantibodies profile in 20 long-term T1D patients used to define percentages of GAD65 pentamer reactive NK cells. (DOCX)

S2 Table GAD65 114–122 selection. Database search of nonamers (A) and decamers (B) of the GAD65 protein sequence with affinity binding to HLA A*02:01. Peptide GAD65 114–122 has high affinity binding. The peptide listed in second position in A was chosen for its high affinity binding respect to the first one (GAD65 141–149) because GAD65 114–122 has the same sequence as decamer 114–123 (B), but without the terminal valine, and its biological significance has been demonstrated [62]. Peptide GAD65 114–123 has low affinity binding (35.01 score), indicating that the subtraction of the terminal valine in GAD65 114–122 plays a key role in the presentation of the motif [38]. Consistently nonamer 115–123 MNILLQYVV having the same sequence than GAD65 114–123 without the initial valine has instead low affinity binding (score 0.316).
(DOCX)
