# Supplementary figures and images for: Identification of GAD65 AA 114-122 reactive 'memory-like' NK cells in newly diagnosed Type 1 diabetic patients by HLA-class I pentamers
Source: PLoS One. 2017 Dec 13;12(12):e0189615. doi: 10.1371/journal.pone.0189615 (PMC5728516; doi:10.1371/journal.pone.0189615)

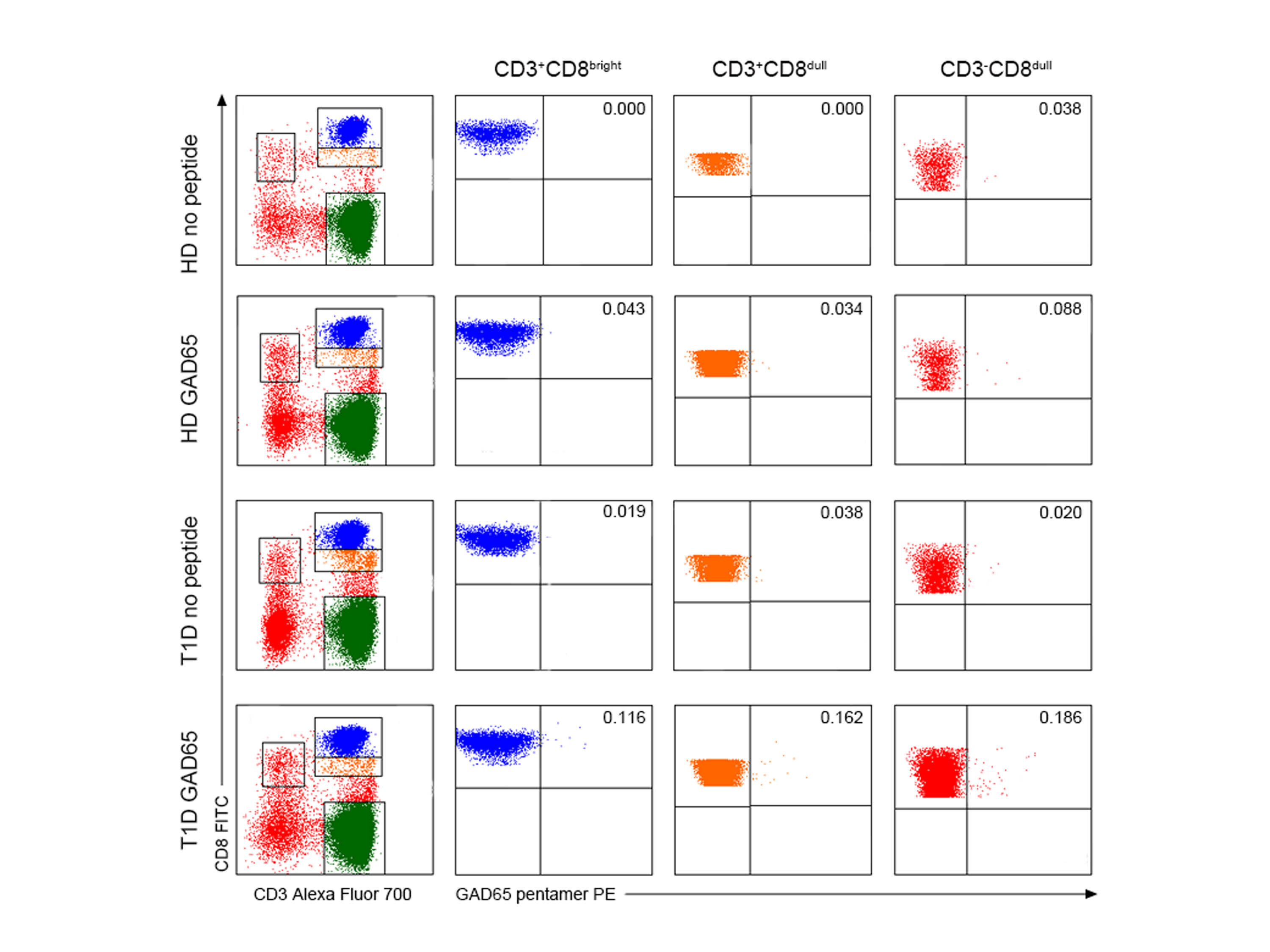

Supplement: S1 Fig — Representative dot plot analysis showing GAD65 AA 114–122 HLA A*02:01 pentamer reactivity on peripheral blood lymphocytes of a T1D patient and a healthy control gated on CD3+CD8bright, CD3+CD8dull and CD3-CD8dull cells. The percentage of GAD65 AA 114–122 pentamer reactivity is indicated for each condition. (TIF) [file pone.0189615.s001.tif]

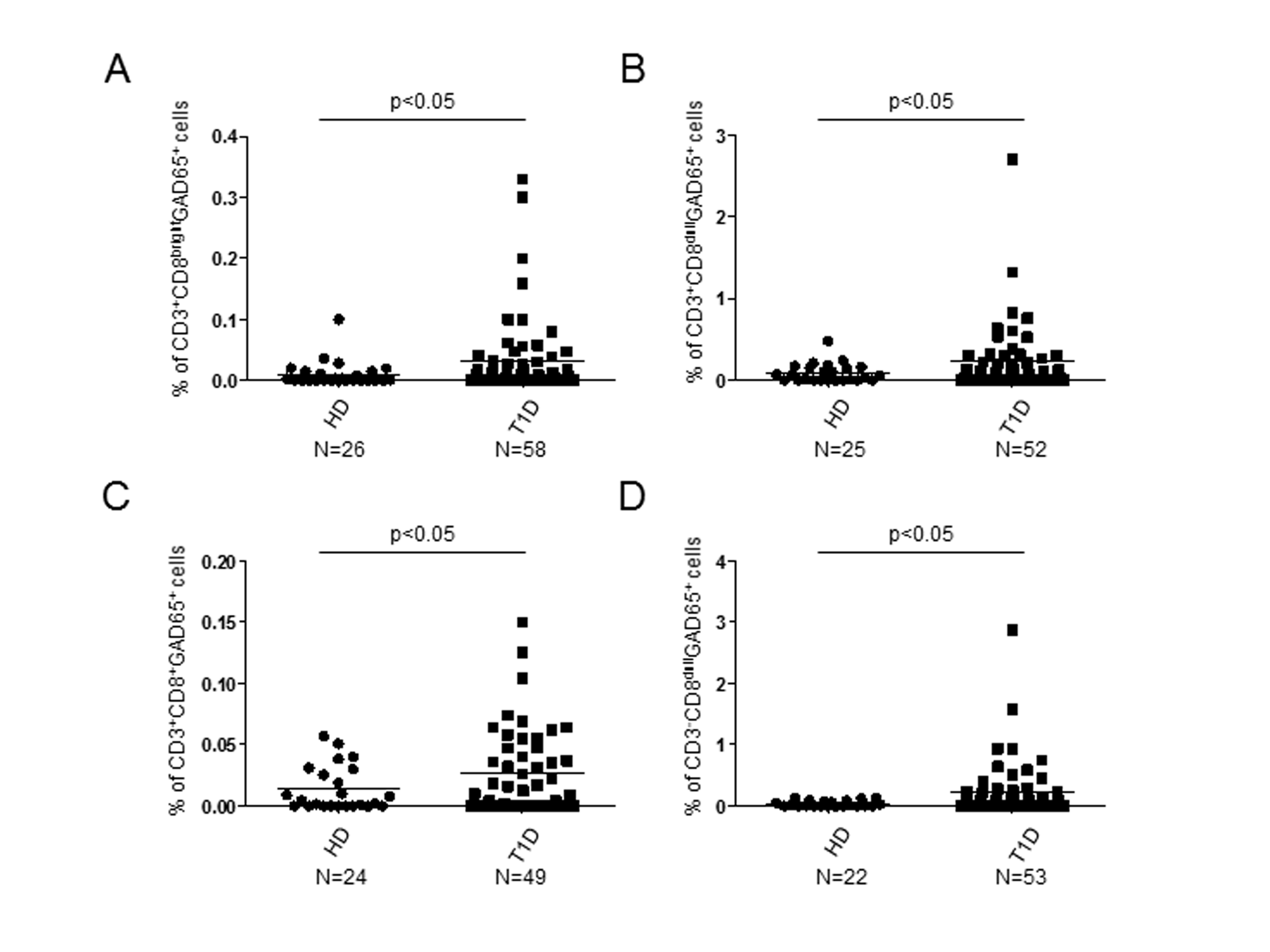

Supplement: S2 Fig — Relative frequencies of CD3+CD8bright (A), CD3+CD8dull (B), total CD3+CD8+ (C) and CD3-CD8dull (D) GAD65 AA 114–122 pentamer reactive cells in T1D patients (square dots) vs healthy controls (circle dots) after GAD65 AA 114–122 peptide stimulation; horizontal bars, average values are reported. Percentages refer to analyzed events within flow-cytometry gates as shown in representative dot plots in S1 Fig. (TIF) [file pone.0189615.s002.tif]

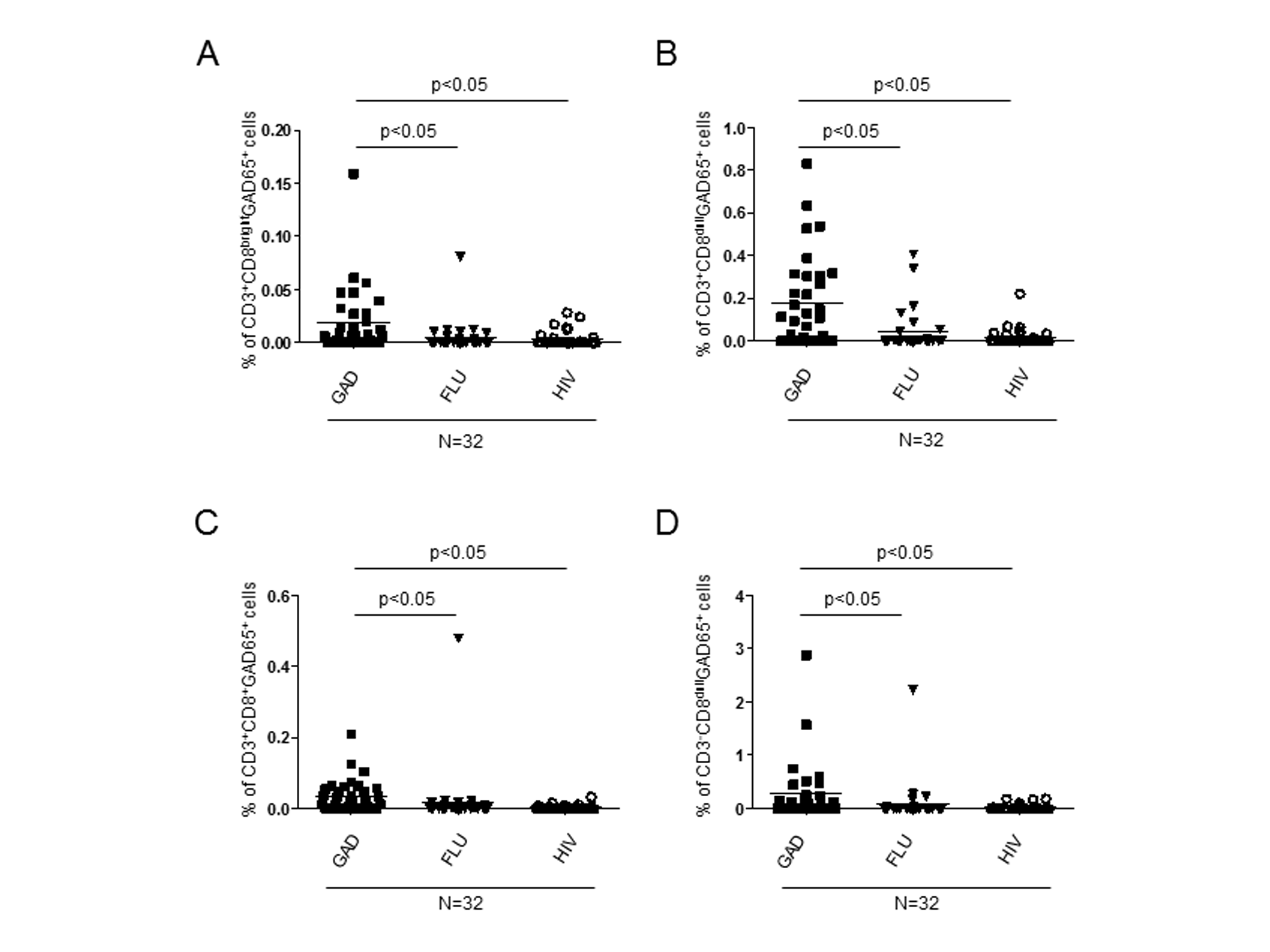

Supplement: S3 Fig — Relative percentages in T1D PBMC of CD3+CD8bright (A), CD3+CD8dull (B), total CD3+CD8+ (C) and CD3-CD8dull (D) GAD65 AA 114–122 pentamer reactive cells after stimulation with GAD65 AA 114–122 peptide (square dots) vs FLU (triangle dots) and HIV peptide (open circle dots); horizontal bars, average values are shown. (TIF) [file pone.0189615.s003.tif]

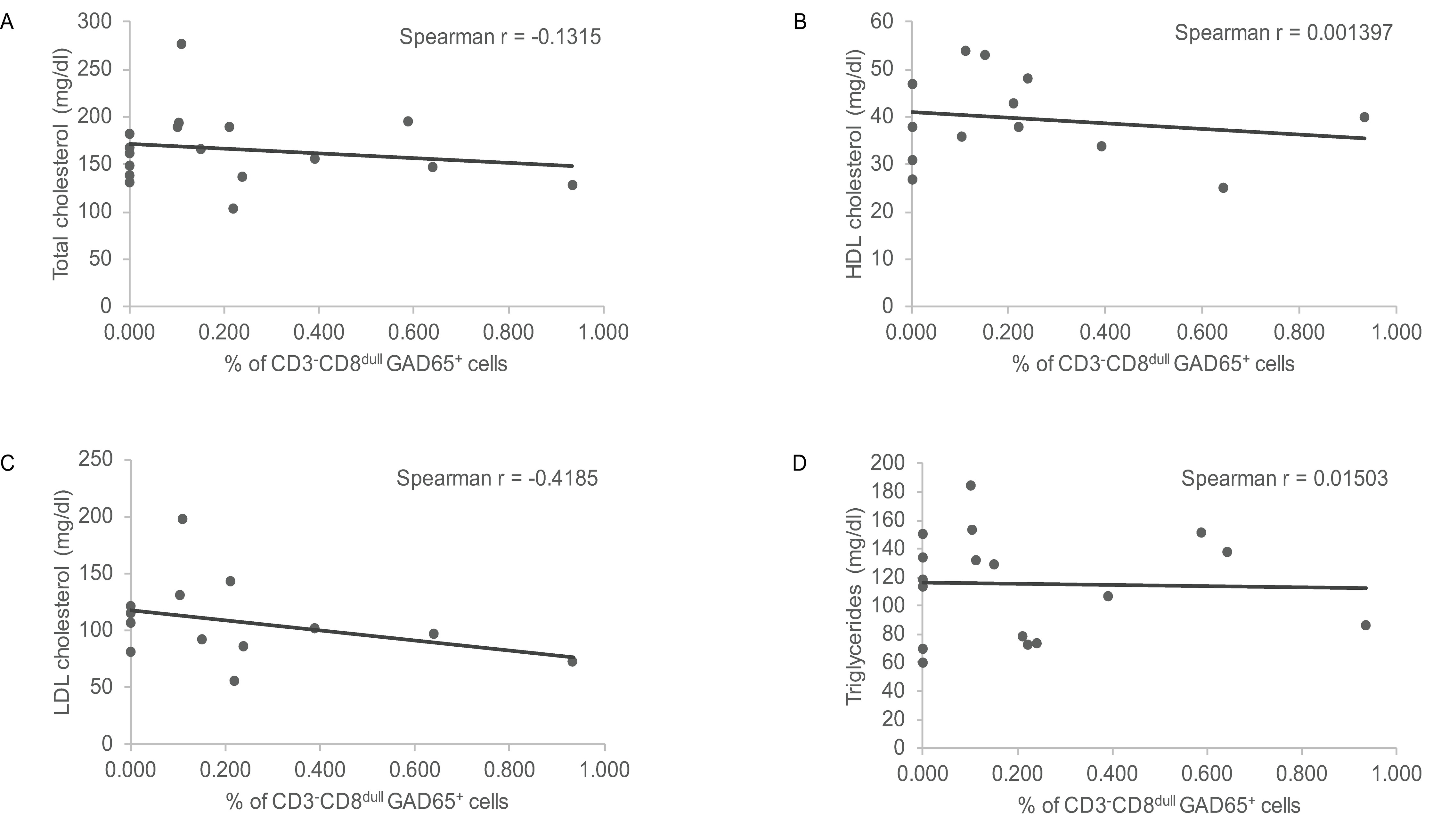

Supplement: S4 Fig — (A) No correlation with total cholesterol levels; (B) No correlation with HDL levels; (C) No correlation with LDL levels; (D) No correlation with triglycerides levels. (TIF) [file pone.0189615.s004.tif]
