# Supplementary material for: Identification of GAD65 AA 114-122 reactive 'memory-like' NK cells in newly diagnosed Type 1 diabetic patients by HLA-class I pentamers
Source: PLoS One. 2017 Dec 13;12(12):e0189615. doi: 10.1371/journal.pone.0189615 (PMC5728516; doi:10.1371/journal.pone.0189615)
Supplement: S1 Table — (DOCX) [file pone.0189615.s005.docx]

| Long term T1D patient | Sex | Age at onset (years,months) | GAD65 Abs (<1U/ml) | IA2 Abs (<1.1U/ml) | IAA (<7%) |
| --- | --- | --- | --- | --- | --- |
| 1^ | F | 12.1 | **9.3** | 0.1 | **25** |
| 3^ | F | 6.9 | NT | NT | NT |
| 4^^a^ | M | 10.6 | **13** | **1.3** | **43** |
| 12^^b^ | F | 13.0 | **39** | **7** | **19** |
| 13^^a^ | F | 10.2 | **2.8** | **4.3** | **18** |
| 15^ | F | 11.0 | 0.7 | **3** | **22** |
| 21^ | F | 12.7 | **3.8** | NT | **9** |
| 22^ | F | 14.2 | NT | NT | NT |
| 25 | M | 5.8 | 0.1 | **1.6** | **12** |
| 27 | M | 9.9 | 0.1 | **7.6** | **19** |
| 32^a^ | M | 12.5 | **97** | **4.3** | **7.2** |
| 33 | F | 8.8 | **49** | **14** | **26** |
| 34 | F | 10.5 | 0.2 | 0.8 | **13** |
| 35 | M | 14.5 | **55** | **2.1** | **20** |
| 36 | M | 12.8 | 0.5 | **6.6** | **38** |
| 37 | M | 12.9 | NT | NT | NT |
| 38 | F | 11.0 | NT | NT | NT |
| 39^a^ | M | 5.6 | NT | NT | NT |
| 40 | M | 10.11 | **6.2** | **27** | **14** |
| 41^b^ | M | 8.40 | NT | NT | NT |

^ tested at onset (see Table 1)

^a^ associated thyroiditis; ^b^ associated thyroiditis and celiac disease
