# Supplementary material for: Identification of GAD65 AA 114-122 reactive 'memory-like' NK cells in newly diagnosed Type 1 diabetic patients by HLA-class I pentamers
Source: PLoS One. 2017 Dec 13;12(12):e0189615. doi: 10.1371/journal.pone.0189615 (PMC5728516; doi:10.1371/journal.pone.0189615)
Supplement: S2 Table — Database search of nonamers (A) and decamers (B) of the GAD65 protein sequence with affinity binding to HLA A*02:01. Peptide GAD65 114–122 has high affinity binding. The peptide listed in second position in A was chosen for its high affinity binding respect to the first one (GAD65 141–149) because GAD65 114–122 has the same sequence as decamer 114–123 (B), but without the terminal valine, and its biological significance has been demonstrated [62]. Peptide GAD65 114–123 has low affinity binding (35.01 score), indicating that the subtraction of the terminal valine in GAD65 114–122 plays a key role in the presentation of the motif [38]. Consistently nonamer 115–123 MNILLQYVV having the same sequence than GAD65 114–123 without the initial valine has instead low affinity binding (score 0.316). (DOCX) [file pone.0189615.s006.docx]

**A**

*Estimate of half time disassociation of a molecule containing this subsequence

SEQ ID No = sequence identification number

**B**
